# Supplementary material for: An unexpected diversity of powdery mildew species infecting the Fabaceae in Australia
Source: PLoS One. 2025 May 15;20(5):e0323505. doi: 10.1371/journal.pone.0323505 (PMC12080826; doi:10.1371/journal.pone.0323505)
Supplement: Attachment — Submitted filename: Response to Reviewers.docx [file pone.0323505.s002.docx]

**Manuscript PONE-D-24-54989**

**Response to Reviewer’s**

Dear Dr Pandey,

We thank you for providing us the opportunity to resubmit a revised draft of the manuscript “An unexpected diversity of powdery mildew species infecting the Fabaceae in Australia” for publication in the Journal PLoS ONE. We appreciate the time and effort that you and the reviewers have dedicated to providing feedback on our manuscript. We are grateful for the valuable comments and have incorporated almost all suggestions in the revised manuscript. Please find my response to the reviewer’s comments below. All page numbers refer to the revised manuscript file with tracked changes.

Kind regards,

Lisa Kelly

**Reviewer’s comments to the authors:**

1. Please ensure that your manuscript meets PLOS ONE's style requirements, including those for file naming. The PLOS ONE style templates can be found at
   <https://journals.plos.org/plosone/s/file?id=wjVg/PLOSOne_formatting_sample_main_body.pdf> and 
   <https://journals.plos.org/plosone/s/file?id=ba62/PLOSOne_formatting_sample_title_authors_affiliations.pdf>

**Author response:** The manuscript meets PLOS ONE’s style requirements.

2. In your Methods section, please provide additional information regarding the permits you obtained for the work. Please ensure you have included the full name of the authority that approved the field site access and, if no permits were required, a brief statement explaining why.

**Author response:** No permits were required to undertake this study and complied with all relevant regulations.

3.	Please review your reference list to ensure that it is complete and correct. If you have cited papers that have been retracted, please include the rationale for doing so in the manuscript text, or remove these references and replace them with relevant current references. Any changes to the reference list should be mentioned in the rebuttal letter that accompanies your revised manuscript. If you need to cite a retracted article, indicate the article’s retracted status in the References list and also include a citation and full reference for the retraction notice.

**Author response:** To our knowledge, all references listed are complete and correct.

**Additional Editor Comments:**

The MS is well organized and written—indetails; however some minor corrections are required before acceptance. I have mentioned some comments on the MS that authors can find useful in revision.

**Author response:** Thank you! We have incorporated almost all suggestions and corrections in the revised manuscript.

The number of Fabaceae species has been clarified in the Abstract on line 27. Additional sentences were added to the Abstract in lines 27 to 30 to provide further detail on the results of the study.

In regards to the number of genera and species that comprise the Fabaceae on line 35, we have kept the original numbers as “640 genera and 18000 species” as that is what is listed in the cited reference (<https://www.sciencedirect.com/science/article/abs/pii/B0121451607001988?via%3Dihub>).

Forage legumes have been added to line 38.

The authority name for each plant species has been listed in lines 41 to 44.

**Reviewers' comments:**

Reviewer #1: Line 63: I would consider adding a few sentences describing the life cycle of powdery mildews on the fabaceae, if a more or less general life cycle is available. This will help the uniformed reader to better understand the importance of the sexual stage, and also why alternative hosts of powdery mildew can be important contributors to disease (lines 82-86). Also include the importance of heterothallic species causing powdery mildew in terms of development of new genotypes of the pathogen(s).

**Author response:** As suggested by the reviewer, we have added a brief description of the asexual and sexual powdery mildew life cycles on lines 63-72.

Line 93: move comma after "including" to after "general".

**Author response:** Done. The comma has now been moved to after “general” on line 108.

Line 105: Delete "crop"

**Author response:** As suggested, we have removed the word, “crop” on line 120.

Line 126: A concern here is if single-conidium isolates was obtained. No mention is made if this was done and if so, how. It is, however, important to obtain single-conidium isolates (different methodologies have been described in literature, given that it is an obligate pathogen), but it’s the only way to ensure that species identification was done using genetically pure specimens.

**Author response:** Our studies, just like many other similar works (e.g., those cited in the manuscript: see references nos. 12-23; 29-30; 35-38; and 44-49) were based on powdery mildew samples freshly collected from the field or preserved as herbarium specimens in internationally recognised collections.

Species identifications were based on these field or herbarium samples (i.e., populations of the respective powdery mildew fungi) based on (a) morphological characteristics that readily distinguish Podosphaera and Erysiphe species (see Figs. 1-4 and the text); and (b) ITS sequences that are always different in different powdery mildew species as revealed, for example in the papers cited in the manuscript and listed above.

**We are not aware of a single publication that used single single-conidium isolates for the identification of powdery mildew species.** This is simply not needed for this purpose and would have been impossible for example in the case of the dried (dead) herbarium specimens used in this work and other publications mentioned above. There is no indication in the literature that morphological patterns and/or ITS sequences would differ amongst single-conidium isolates of the same species. Intra-specific variation of ITS sequences was detected in a few powdery mildew samples as “exceptions to the rule” (this was done by our group: Kovacs et al. 2011, Eur J Plant Pathol 131: 135-141; and Kiss 2012, PNAS 109: E1811). However, these rare intra-specific differences were detected amongst powdery mildew populations, not single-conidium isolates.

Single-conidium isolates are regularly produced in our laboratory for whole-genome sequencing projects and also when we study for example mating types or fungicide resistance patterns in diverse powdery mildews. In those studies, single-conidium isolates are the standard way of conducting research, whereas species identifications do not require this approach.

Line 241: Why was no chasmothecia observed? Is it an inherent trait of these species that chasmothecia is not readily produced, or was it due to samples being collected prior to formation of chasmothecia? Please add a line of two in the discussion on this topic.

**Author response:** This is an interesting question. Our study supports earlier findings that most powdery mildews do not produce chasmothecia in Australia (see references 15-17 and 19, 20). We propose that the Australian climate allows the continual survival of the asexual stage of most powdery mildew species on their hosts. We have added a few sentences to the discussion on lines 498 to 502.

Line 245: The no of specimens is confusing. Mention is made of 34 fresh specimens in line 171, and when you add up the rest of the specimens (line 171-177) it equals 69 (34 + 17 + 9 + 9). Please clarify.

**Author response:** Thank you for your suggestion. To clarify, there were 34 freshly collected powdery mildew specimens and 40 herbarium specimens as stated in lines 186-188. An additional 19 sequences from recently identified species from Fabaceae hosts were included in the phylogenetic analyses as references: 17 for Erysiphe and 2 for Podosphaera (Figs. 5 and 6). Of the fresh and herbarium specimens, 17 specimens belonged withing the *Vigna* genus, 9 within the *Vicia* genus, and 9 within the *Glycine* genus. Specimens from other genera were not mentioned in the text but are shown in the Tables. All numbers referring to the samples/specimens included in the different parts of this study were double checked in the manuscript. Also, the number of samples/specimens was specified in the revised Abstract (lines 27-30).

Line 254: Mention as a subscript below the table that the species was identified molecularly, so readers won’t be confused about how identification was done.

**Author response:** Great suggestion. I have added the subscript to the table to clarify how the species were identified.

Line 428: Add a fullstop at the end of the sentence.

**Author response:** Done (line 443).
